# Complete Genome Sequence of Salmonella enterica subsp. *enterica* Serovar Kottbus Strain Kharkiv, Isolated from a Commercial Pork Production Facility in Ukraine

**DOI:** 10.1128/MRA.01171-20

**Published:** 2020-12-03

**Authors:** Vasiliy Arefiev, Ganna Kovalenko, Maciej Frant, Tetiana Chumachenko, Yuliia Polyvianna, Svitlana Pivnenko, Vitaliy Bolotin, Olga Mayboroda, Oleksii Solodiankin, Oleksandr Tarasov, Maksym Bezyemenni, Cora Lyon, Matthew Redlinger, Maryna Sapachova, Andrii A. Mezhenskyi, Anne-Lise Ducluzeau, Eric Bortz, Anton Gerilovych, Devin M. Drown

**Affiliations:** a National Scientific Center, Institute of Experimental and Clinical Veterinary Medicine, Kharkiv, Ukraine; b National Academy of Agrarian Sciences of Ukraine, Institute for Veterinary Medicine, Kyiv, Ukraine; c National Veterinary Research Institute, Pulawy, Poland; d Kharkiv National Medical University, State Institution Kharkiv Oblast Laboratory Center of the Ministry of Health of Ukraine, Kharkiv, Ukraine; e Department of Biological Sciences, University of Alaska Anchorage, Anchorage, Alaska, USA; f State Scientific Research Institute of Laboratory Diagnostics and Veterinary and Sanitary Expertise, Kyiv, Ukraine; g Department of Biology and Wildlife, University of Alaska Fairbanks, Fairbanks, Alaska, USA; h Institute of Arctic Biology, University of Alaska Fairbanks, Fairbanks, Alaska, USA; University of Maryland School of Medicine

## Abstract

The complete genome of Salmonella enterica subsp. *enterica* serovar Kottbus strain Kharkiv (serogroup C2-C3), which was isolated from a commercial pork production facility in Kharkiv, Ukraine, was assembled using long-read Nanopore sequences. A single circular contig (4,799,045 bp) comprised a complete chromosome encoding antibiotic resistance, highlighting the risk of cross-species livestock and human infection.

## Abstract (precis)

The complete genome of Salmonella enterica subsp. *enterica* serovar Kottbus (serogroup C2-C3), which was isolated from a commercial pork production facility in Kharkiv, Ukraine, was assembled using long-read Nanopore sequences. A single circular contig (4,799,045 bp) comprised a complete chromosome encoding antibiotic resistance, highlighting the risk of cross-species livestock and human infection.

## ANNOUNCEMENT

As part of a scientific-capacity-building initiative in Ukraine to better understand African swine fever outbreaks, the genomes of concurrent or coinfecting pathogens of commercial and backyard swine and wild boar were sequenced in regional veterinary laboratories. For this study, the original sample source was a commercial pork production facility (chopping boards) identified by the State Institution Kharkiv Oblast Laboratory Center of the Ministry of Health of Ukraine. A *Salmonella* sp. strain was isolated on 6 June 2018 from the Sakhnovshchanskyi Rayon district of Kharkiv Oblast (province). The primary enrichment of the microbial culture was carried out using selenite broth. After 1 day of incubation, parallel inoculation was carried out on Endo agar and bismuth sulfite agar (37°C). On 5 July 2018, the strain was transferred to the National Scientific Center Institute of Experimental and Clinical Veterinary Medicine (IECVM) bacteriological laboratory. A tryptic soy broth (TSB) culture grown from a single colony incubated overnight at 22°C was cryopreserved at −70°С in cryovials at IECVM prior to DNA isolation. To obtain DNA for sequencing, we inoculated liquid cultures of TSB, incubated them overnight at 22°C, and used 1.8 ml as input for the DNeasy UltraClean microbial kit (Qiagen).

We used 1 μg of DNA as input for a rapid sequencing library (SQK-RAD004; Oxford Nanopore Technologies [ONT]) and sequenced it on an R9.4.1 flow cell (FLO-MIN106, flow cell identification no. FAK94576) for 26 h using a MinION Mk1B device (ONT). We base called raw data with Guppy v3.6.1 (ONT) using the high-accuracy model (c, dna_r9.4.1_450bps_hac.cfg) and default parameters. This run generated a total of 9,547,083,284 bp in 3,536,512 reads with a read length *N*_50_ value of 5,727 bp. We used Filtlong v0.2.0 (https://github.com/rrwick/Filtlong) to filter by a length of ≥1,000 bp (min_length, 1000) and quality (Q) score of ≥10 (min_mean_q, 90). After filtering, we had 7,261,568,727 bp in 1,612,614 reads with a read length *N*_50_ value of 6,283 bp and a median Q score of 13.4.

We assembled quality-controlled read data *de novo* using Flye v2.7 (1) with default parameters, specifying the estimated genome size (-genomesize=5m), Nanopore reads (-nanopore-raw), and subsampling for initial disjointing assembly (--asm-coverage 100). We corrected the single contig, marked as circular by Flye, using two rounds of Racon v1.3.1 (2) polishing with the following parameters: score for matching bases: match, 8; score for mismatching bases: score for matching bases, --match 8; score for mismatching bases, --mismatch -6; threshold for average base quality of windows, --quality-threshold -1; default gap penalty, --gap -8; and default window --window-length 500. We ran a final polish with Medaka v1.0.3 (https://github.com/nanoporetech/medaka) specifying the base-caller model (-m r941_min_high_g360) and using default parameters. For polishing, we used the entire 7.2-Gb quality-controlled data set (coverage, 970×). Our 4,799,045-bp polished assembly (*N*_50_, 4,799,045 bp) consists of one circular contig (GC content, 52.21%).

To annotate the genome, we used PATRIC v3.6.5 (3, 4) to assign 84 tRNAs, 22 rRNAs, 104 repeat regions, and 4,870 coding sequences. PATRIC reported a 100% completeness score with minor contamination (1.6%) (5). PATRIC identified a total of 57 antibiotic resistance genes (Table 1) and 232 virulence factors. PATRIC assigned the isolate as a member of the Salmonella enterica group with multilocus sequence typing (MLST) sequence type 212. Using the *Salmonella in Silico* Typing Resource (SISTR) command line tool (6), the genome was assigned to serogroup C2-C3 and serovar Kottbus. SISTR provided O-antigen (types 6 and 8) and H-antigen (types e and h and types 1 and 5) types consistent with determined serology. We assign this isolate as Salmonella enterica subsp. *enterica* serovar Kottbus strain Kharkiv. The genome deposited in GenBank was annotated with PGAP (7) during submission.

**TABLE 1:** Antimicrobial resistance genes

Antimicrobial resistance mechanism	Gene(s) or gene product(s)
Antibiotic activation enzyme	KatG
Antibiotic inactivation enzyme	AAC(6')-Ic, AAC(6')-If, AAC(6')-Ig, AAC(6')-Ih, AAC(6')-Ij, AAC(6')-Ik, AAC(6')-Il, AAC(6')-Ir, AAC(6')-Is, AAC(6')-It, AAC(6')-Iu, AAC(6')-Iv, AAC(6')-Iw, AAC(6')-Ix, AAC(6')-Iy, AAC(6')-Iz
Antibiotic resistance gene cluster, cassette, or operon	MarA, MarB, MarR
Antibiotic target in susceptible species	Alr, Ddl, *dxr*, EF-G, EF-Tu, *folA*, Dfr, *folP*, *gyrA*, *gyrB*, *inhA*, *fabI*, Iso-tRNA, *kasA*, MurA, *rho*, *rpoB*, *rpoC*, S10p, S12p
Antibiotic target protection protein	BcrC
Efflux pump conferring antibiotic resistance	AcrAB-TolC, AcrAD-TolC, AcrEF-TolC, AcrZ, EmrAB-TolC, MacA, MacB, MdfA/Cmr, MdtABC-TolC, MdtL, MdtM, MexPQ-OpmE, OprM/OprM family, SugE, TolC/OpmH
Gene conferring resistance via absence	*gidB*
Protein altering cell wall charge, conferring antibiotic resistance	GdpD, PgsA
Regulator modulating expression of antibiotic resistance genes	AcrAB-TolC, EmrAB-TolC, H-NS, OxyR

### Data availability.

This whole-genome shotgun project has been deposited in GenBank under the accession no. CP062220. The version described in this paper is the first version, CP062220.1. Raw data for this project can be found in the GenBank SRA under PRJNA666173.
